# Renal artery aneurysm induced by neurofibromatosis type 1: A case report and review of the endovascular interventions for this rare vasculopathy

**DOI:** 10.1097/MD.0000000000034216

**Published:** 2023-07-07

**Authors:** Sultan AlSheikh

**Affiliations:** a Division of Vascular Surgery, Department of Surgery, College of Medicine, King Saud University, Riyadh, Saudi Arabia.

**Keywords:** endovascular interventions, neurofibromatosis type 1, renovascular hypertension, renal artery aneurysm

## Abstract

The autosomal dominant condition known as neurofibromatosis type 1 (NF-1) is characterized by café au lait macules and neurofibromatosis. Aneurysms in renal arteries are rare. Renal artery aneurysm (RAA) can be successfully treated with endovascular procedures; however, successful cases in NF-1 adults have not been reported.

**Patient concerns:**

Here, we report the case of a 30-year-old female suffering from NF-1. The patient presented to the emergency department with complaints of chronic, poorly controlled hypertension. A left RAA was found om the computed tomography angiography (CTA).

**Diagnoses:**

A left renal artery aneurysm was diagnosed using CTA during workup for secondary hypertension.

**Interventions:**

Selective angiographym of the left renal artery confirmed a fusiform aneurysm of the distal renal artery. A self-expandable covered stent was placed, and a completion angiogram demonstrated good aneurysm sealing and contrast flow to the left kidney.

**Outcomes:**

The patient’s blood pressure improved after the procedure. Her medications were lowered to almost half of their baseline doses, and hydralazine was discontinued. On the follow-up visit after 4 months, the patient reported his home-measured systolic blood pressure to be less than 120 mm Hg. A repeated CTA of the abdomen showed post-left RAA repair with a covered stent and interval improvement of the left kidney.

**Lessons:**

RAA caused by NF-1 are manageable and feasible with endovascular intervention.

## 1. Introduction

Neurofibromatosis type 1 (NF1) is an autosomal dominant genetic disorder, also known as von Recklinghausen disease. This condition results from a mutation in NF1 gene, which is localized on chromosome 17q11.2.^[1]^ NF1 clinical characteristics vary from patient to patient, and the most common symptoms include the development of multiple non-cancerous tumors on nerve tissue, called neurofibromas, malignant peripheral nerve sheath tumors, scoliosis, tibial dysplasia, vasculopathy, and pulmonary disease.^[2]^ Patients with NF1 are at an increased risk of developing hypertension, with an incidence rate approximately 16%.^[3]^ Hypertension can be essential or secondary to coarctation of the aorta, renovascular disease, or phaeochromocytoma.^[2,4]^ The cardiovascular features of NF1 may include stroke or transient ischemic attack, coronary artery disease, retinal artery occlusion, peripheral vascular disease, carotid and renal artery stenosis, and in some cases, vasculopathy can even lead to aneurysm formation.^[5]^ NF1 is a common cause of renal artery disease, but most cases are stenotic lesions rather than aneurysms.^[6,7]^ Focusing on the treatment of the symptoms’ presentation is the current management approach for NF1 patients. As a result, early detection of renal artery aneurysm (RAA) is crucial because curative treatment can eliminate the adverse effects of hypertension. Endovascular treatment of RAA associated with NF-1 has rarely been reported. The author reports a case of an adult female NF-1 patient who presented with hypertension and RAA and was treated successfully by an endovascular procedure.

## 2. Case report

A 30-year-old female was diagnosed with NF-1. The patient presented to the emergency department of the referring hospital with complaints of chronic, poorly controlled hypertension with a blood pressure of approximately 171/106 mm Hg while taking methyldopa 500 mg 4 times daily and hydralazine 25 mg 4 times daily. The patient was admitted and started on labetalol 200 mg twice daily, in addition to her previous antihypertensive medications. She was referred to the endocrinology unit for assessment of secondary hypertension. A workup was performed to rule out a pheochromocytoma in the context of neurofibromatosis. In addition, primary hyperaldosteronism, renovascular hypertension, coarctation of the abdominal aorta, thyroid and parathyroid disease, and Cushing’s disease were investigated.

**Figure 1.**
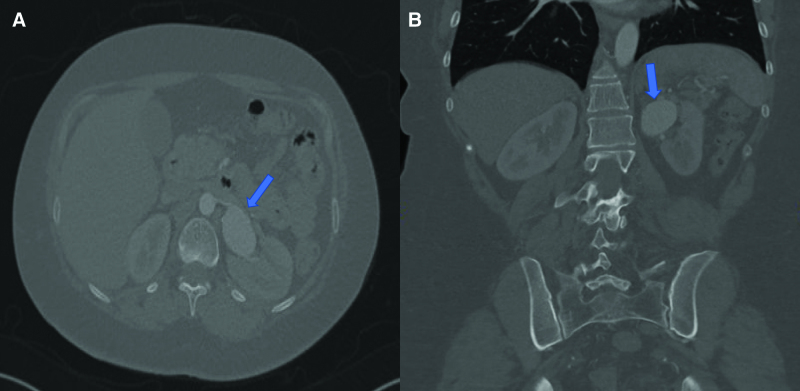
(A) Computed tomography angiography of the renal arteries axial view showed a fusiform aneurysm of the distal left renal artery measuring 4.1 × 2.7 × 4 cm with peripheral calcifications (arrowhead). (B) Coronal view of the fusiform aneurysm of the distal left renal artery.

**Figure 2.**
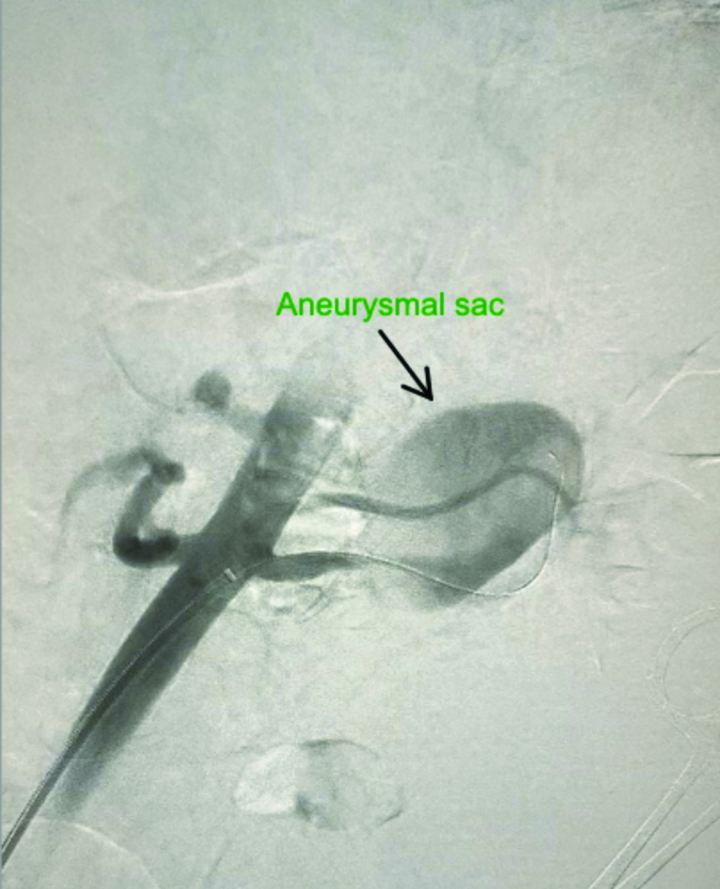
Selective angiogram of the left renal artery confirmed a fusiform aneurysm of the distal renal artery (arrowhead).

**Figure 3.**
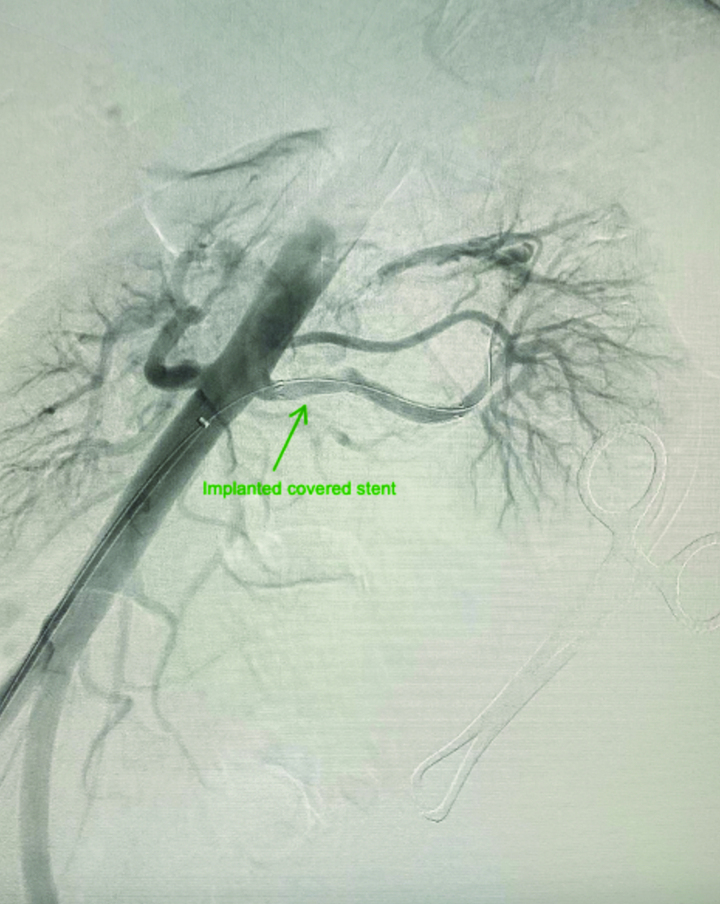
Completion angiogram of the left renal artery showed a covered stent in place with good aneurysm sealing (excluding the flow into the aneurysm) and no evidence of endoleak or extravasation (arrowhead).

**Figure 4.**
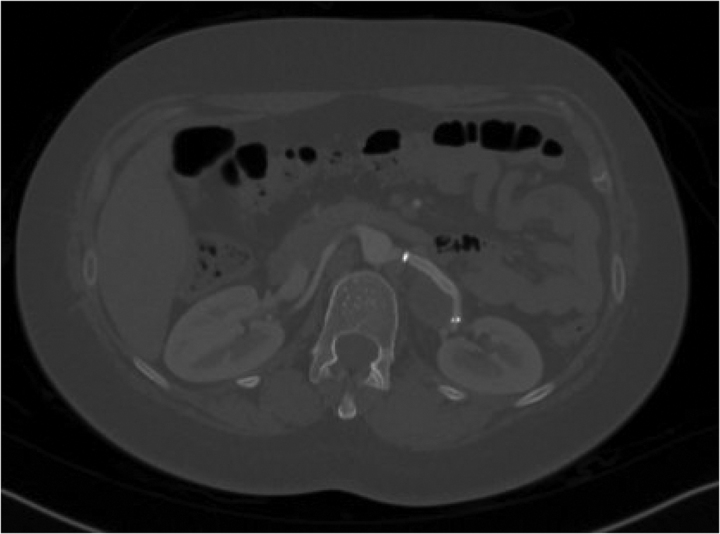
Postoperative computed tomography scan of the renal arteries axial view showed a post left renal artery aneurysm repair with a covered stent and a completely thrombosed aneurysm without extravasation or endoleak.

Complete blood cell count, routine urinalysis, blood chemistry profile (potassium, sodium, creatinine, fasting glucose, and fasting lipid levels), plasma and 24 urine metanephrine, aldosterone to renin ratio, and 24 urine cortisol tests were all came within normal ranges. Computed tomography angiography (CTA) of the abdomen revealed a fusiform aneurysm of the distal left renal artery measuring 4.1 × 2.7 × 4 cm with peripheral calcifications (type I RAA) (Fig. 1). No intramural thrombus, perianeurysmal fluid, or active contrast extravasation was detected. No stenosis was seen in the ostium of the left renal artery. The right renal artery was unremarkable with no evidence of stenosis or aneurysm. No other aneurysms or vascular malformations were seen on the abdomen. Given the possible need for vascular intervention, the patient was transferred to our vascular surgery service. We discussed the options with the patient, including open surgical reconstruction or endovascular intervention. The decision was made to perform endovascular intervention by placing a covered stent based on the type of RAA that was seen in her preoperative CTA. A selective angiogram of the left renal artery confirmed a fusiform aneurysm of the distal renal artery (Type I RAA) (Fig. 2). We were able to pass the aneurysm with a wire and catheter, and a 6-mm X 25-mm polytetrafluoroethylene (PTFE)-covered GORE VIABAHN (W. L. Gore & Associates, Inc, Newark, Delaware, AZ) was placed. A completion angiogram demonstrated good aneurysm sealing (excluding the flow into the aneurysm) without evidence of endoleak or extravasation and good contrast flow to the left kidney (Fig. 3). Postoperative recovery was uneventful, and the patient was kept on aspirin and clopidogrel. A repeated CTA of the abdomen showed a post-left renal artery aneurysm repair with a covered stent with no flow seen in the aneurysm. The patient was discharged home on postoperative day 1 with regular outpatient clinic follow-up. On a follow-up visit with her primary endocrinologist after 2 months, it was noted that the patient had improved blood pressure. Her medications were lowered to almost half of their baseline doses (methyldopa was reduced to 250 mg twice daily, and labetalol was reduced to 100 mg twice daily). Hydralazine was discontinued. On a follow-up visit after 6 months, a repeated CTA of the abdomen showed a post-left renal artery aneurysm repair with a covered stent and a completely thrombosed aneurysm without extravasation or endoleak (Fig. 4). The patient reported a home-measured systolic blood pressure of <120 mm Hg. The clopidogrel was discontinued, and she was kept on aspirin.

## 3. Discussion

NF1 involves the development of café-au-lait spots on the skin, freckling in the armpits and groin area, and neurofibromas.^[8]^ The wide range of characteristics NF1 exhibits shows how severe the condition is and its exponential impact on human health, as it lowers the quality of life. Several criteria have been used for the diagnosis of NF1. All these criteria focused on providing a better understanding of disease patterns. For instance, the revised diagnostic criteria for NF1 include the presence of two or more of the following: six or more café-au-lait spots; two or more neurofibromas, freckling in the axillary or groin area, two or more Lisch nodules of the iris, a first-degree relative (parent, sibling, or child) with NF1; or an optic glioma.^[9]^ Moreover, some people with NF1 may develop cardiovascular disease, which is known as NF-1 vasculopathy.^[6]^ NF1 is a common cause of renal artery disease, but most cases are stenotic lesions rather than aneurysms.^[6,7]^ Diagnosis of RAA usually involves a combination of imaging studies and clinical evaluations. CTA is the imaging modality most frequently used to assess renal artery aneurysms, followed by non-contrast enhanced computed tomography, magnetic resonance angiography, catheter angiography, and ultrasonography.^[10]^ In order to assess patients with a suspected RAA, the current vascular surgery guidelines recommend using CTA as the first imaging modality of choice.^[11]^ The angiographic classification of the RAA according to their location is as follows^[12]^: Type I saccular aneurysms originate either from the main renal artery or proximally from a major segmental branch (this is the most common type). Type II fusiform/saccular aneurysms arise either at the main renal artery or at the proximal segmental branch (after renal artery bifurcation). Type III—intra-lobar aneurysms originate either from accessory arteries or small segmental arteries (arising from the intraparenchymal renal artery).

The Society for Vascular Surgery clinical practice guidelines on the management of visceral aneurysms published in 2020^[11]^ recommended a set of criteria for treating the renal artery aneurysms, including the following: an aneurysm larger than 3 cm. An aneurysm less than 3 cm with any of the following: hypertension, female of childbearing age or pregnant female, hematuria, deteriorating renal function, flank pain, patients with a single kidney, dissecting aneurysms causing stenosis, infraction growing intrarenal, or thromboembolism. An aneurysm rupture. The current guidelines have increased the size threshold for interventions to >3 cm for asymptomatic aneurysms, compared to the previous size threshold of >2 cm for repair of visceral artery aneurysms, due to the extremely slow growth rates and 3% to 5% rupture rates in recently published studies.^[11]^

### 3.1. Endovascular management entails various techniques depending on the type and location of the aneurysm

#### 3.1.1. Type I renal artery aneurysm endovascular treatment options.

##### 3.1.1.1. Endoprosthesis/covered stents.

The most commonly covered stents used in the literature are PTFE-covered GORE VIABAHN (W. L. Gore & Associates, Inc)^[13]^ endoprosthesis with heparin bioactive surface, an autologous saphenous vein-covered stent,^[14]^ Palmaz stents (Cardinal Health, Dublin, Ohio, UK),^[12]^ and PTFE covered Jostent peripheral stent graft.^[15,16]^ Nitinol and PTFE are the most commonly used materials in covered stents. These stents are used to treat aneurysms located up to 15 mm from the ostium of the renal artery to provide a sufficient landing zone. If the aneurysm is located more distally, the renal artery can be stented all the way to the ostium and coils can be placed into the aneurysmal sac. The advantage of this approach is that it can simultaneously treat renal artery stenosis and exclude aneurysms in the same procedure.^[17]^ The diameter and length of the stent are measured based on the CTA and pre/intraoperative catheter angiogram images. These stents are not suitable for us in bifurcations, type II RAA because of the risk of covering important branches, and type III RAA because of flexibility challenges while navigating through the smaller caliber tortuous distal branches.

##### 3.1.1.2. Selective embolization with coils.

This is the most often recommended approach for treating saccular aneurysms with a neck size less than 4 mm in diameter or an aneurysm-to-neck ratio >2:1.^[13]^ This approach can be used to treat all 3 types of RAA. This option is not recommended to be used in aneurysms of wide-neck and complex aneurysms with efferent branches.

#### 3.1.2. Type II renal artery aneurysm endovascular treatment options.

This type of aneurysm presents a therapeutic challenge for endovascular interventions due to the location and the complex anatomy of the aneurysm. This increases the potential risk of coil protrusion into the main artery and reduces the rate of complete occlusion. Therefore, nephrectomy and open vascular surgical procedures such as extracorporeal arterial reconstruction and autotransplantation are still considered the gold standard approaches for treating this type of aneurysm.

##### 3.1.2.1. Multilayered endoprosthesis and flow diverter stents.

This procedure aims to decrease the turbulent flow within the aneurysm while improving laminar flow in the arterial system. These stents have intertwined layers in their structure, which will help to achieve this goal by limiting the flow and encouraging thrombosis in the aneurysm sac. For RAA, 2 specific stents were used: the Surpass flow diverter stent (Surpass; Stryker Neurovascular, Kalamazoo, MI) and Cardiatis Multilayer Stent (Cardiatis, Gembloux, Belgium).^[18]^ When treating complex aneurysms with many efferents, the flow diverter is passed through the efferent artery with the largest diameter, redirecting native arterial flow and decreasing flow through other efferents (also known as jailed branches) and within the aneurysm sac. As a result, the sac will gradually thrombose, and the stent mesh becomes endothelialized. Due to the possibility of in-stent stenosis, dual antiplatelet medication is recommended over the long term.^[18]^ The flow diverter is not suitable because of the flexibility challenges and the smaller profile required to treat type III RAA.

##### 3.1.2.2. Remodeling techniques

Using balloon-expandable/self-expandable non-covered stents and microcoils, this method is frequently used to treat complicated aneurysms, including type I and type II. Following selective catheterization, the stent is placed over the aneurysm neck and used as a support to deliver a microcatheter through its mesh. Through this catheter, microcoils are introduced into the aneurysm sac, changing the local flow pattern, and later the stent mesh goes through endothelialization. Alternatively, if the aneurysm base to sac ratio is less than 70% and the proximal artery is thin, coil placement can be performed first, followed by stent placement.^[19]^ In anatomically appropriate locations with a low profile, the remodeling technique can also be performed with catheterization and the deployment of microcoils without stent placement. Balloon-assisted coil embolization is a procedure that involves inflating the balloon at the aneurysm neck to achieve remodeling of the anatomy, inserting a microcatheter tip into the sac, and then placing microcoils.^[20]^ Managing wide neck aneurysms requires using this dual microcatheter technique, one to deploy a balloon and the other to fill the sac. This technique prevents coil migration into the proximal artery by placing an inflated balloon.

#### 3.1.3. Type III renal artery aneurysm endovascular treatment options.

Liquid embolic agents and coil embolization are used for super-selective embolization of the renal segmental branch. Microcatheters are used to deliver liquid embolic agents such as Onyx^®^ (ethylene vinyl alcohol copolymer dissolved in DMSO) (Micro Therapeutics, Inc., Irvine, CA), and N-butyl cyanoacrylate-Hystoacril^®^ (B. Braun Surgical S.A. [Melsungen, Germany] in Spain).^[13]^ To improve the radio-opacity, these agents can be mixed with Lipiodol^®^ or Micronized tantalum powder.

### 3.2. Potential complications, outcome, and follow-up

Myocardial infarction and renal failure requiring dialysis are major perioperative and early postoperative complications, while minor complications include urinary tract infection, urinary retention, puncture site infection, minor kidney infarction, and transient renal insufficiency. Late postoperative complications included stent stenosis, renal artery thrombosis, renal bypass thrombosis, post-embolization syndrome, and chronic abdominal abscess.^[19]^ Ghosh and Dutta^[21]^ analyzed 14 published studies in which 157 RAAs were treated. The most commonly reported complications are renal infarction (15%), postembolization syndrome (leukocytosis, fever, abdominal pain, nausea and vomiting) (5%), coil migration into the native renal artery compromising a significant area of the renal parenchyma (1.2%), and aneurysm sac expansion reperfusion (<1%). In comparison to the operative group, the endovascular group had a shorter operative time, lower blood loss, shorter intensive care unit stay, and shorter hospital stays.^[22,23]^ However, in-hospital mortality in the endovascular group was higher than that in the operative group (1.8% vs 0.9%).^[23]^ In contrast, cardiac (2.2% vs 0.6%) and peripheral vascular complications (0.6% vs 0.0%) were higher in the operative group.^[23]^

The Society for Vascular Surgery clinical practice guidelines on the management of visceral aneurysms published in 2020^[11]^ suggested a set of recommendations for follow-up of RAA, including: CTA or magnetic resonance angiography or arteriography for post-intervention prior to hospital discharge; post-intervention surveillance with imaging at 1 month, 6 months, and then annually untill relevant; and annual surveillance imaging until 2 subsequent studies were stable for patients who were managed conservatively (no intervention).

## 4. Conclusion

Renovascular hypertension and pheochromocytoma should be screened in patients with NF-1 and hypertension. Renal artery aneurysms are considered manageable and feasible with endovascular intervention, which provides patients with excellent technical and clinical outcomes along with shorter operative time, lower blood loss, shorter intensive care unit stay, and shorter hospital stays compared to standard surgery. Type II and type III aneurysms, which previously required surgical management, can now be treated with embolization therapy and flow diversion stents because of advancements in endovascular therapies.

## Author contributions

**Conceptualization:** Sultan AlSheikh.

**Data curation:** Sultan AlSheikh.

**Formal analysis:** Sultan AlSheikh.

**Investigation:** Sultan AlSheikh.

**Writing – original draft:** Sultan AlSheikh.

**Writing – review & editing:** Sultan AlSheikh.
